# Evaluation of the Prognostic Value of Lactate and Acid-Base Status in Patients Presenting to the Emergency Department

**DOI:** 10.7759/cureus.15857

**Published:** 2021-06-23

**Authors:** Ramiro D'Abrantes, Laura Dunn, Tim McMillan, Benjamin Cornwell, Ben Bloom, Tim Harris

**Affiliations:** 1 Anaesthesia, Milton Keynes University Hospital, Milton Keynes, GBR; 2 Emergency Medicine, Barts Health NHS Trust, London, GBR; 3 Emergency Medicine, Mid Yorkshire NHS Trust, West Yorkshire, GBR; 4 Emergency Medicine, Queen Mary University of London, London, GBR

**Keywords:** lactate, lactic acidosis, acid-base status, emergency department, in-hospital mortality

## Abstract

Background

Lactate levels predict mortality in a wide range of patients presenting to the Emergency Department (ED); however, the effect of co-existing acidosis is unknown. Here, we investigated the effect of acidosis on in-hospital mortality for patients with hyperlactataemia.

Methodology

This is a retrospective cohort study of adults cared for in the resuscitation area of one ED who received a metabolic panel on arrival. The primary outcome was in-hospital mortality for patients with normal lactate (0.0-2.0 mmol/L), intermediate lactate (2.1-4.0 mmol/L), or high lactate (>4.0 mmol/L), with and without acidosis. Odds ratios (ORs) were calculated to assess the differences in mortality rates between groups stratified by lactate and acid-base status.

Results

A total of 4,107 metabolic panels were collected and 3,238 were assessed. In total, 510 (15.8%) and 784 (24.2%) patients had a normal lactate and acidosis/no acidosis; 587 (18.1%) and 842 (26.0%) had intermediate lactate and acidosis/no acidosis; and 388 (12.0%) and 127 (3.9%) had high lactate and acidosis/no acidosis, respectively. The overall mortality was 5%. Mortality was 4.3%/0.6% in the normal lactate, 5.6%/2.6% in the intermediate lactate, and 19.3%/3.9% in the high lactate groups, with and without acidosis, respectively. Combining base excess <-6 and lactate >4 mmol/L had a sensitivity of 39%, specificity of 96%, positive predictive value of 32%, and negative predictive value of 98% for in-hospital mortality (OR: 14.0; 95% confidence interval: 9.77-20.11).

Conclusions

In an undifferentiated cohort of ED patients presenting to the resuscitation area lactaemia associated with acidosis is a more accurate predictor of in-hospital mortality than hyperlactataemia.

## Introduction

Early recognition of critical illness in the Emergency Department (ED) is associated with improved outcomes [1,2]. Physiological scores and biochemical measurements (creatinine, acid-base status, and serum lactate) are widely used to identify high-risk patients requiring urgent intervention [3-6]. Point-of-care metabolic panels, most commonly based on blood gas analysis, provide these parameters to clinicians within minutes of venepuncture.

Lactate levels are widely used to identify a critical illness. Three recent international trials and the surviving sepsis campaign have used lactate levels to identify patients in septic shock with no differentiating for venous, central venous, or arterial sampling [7,8]. There is no universally accepted value to define hyperlactataemia, with the upper limit of normal ranging from 1.0 to 2.5 mmol/L [9-13]. Hyperlactataemia is associated with adverse outcomes in undifferentiated ED patients and in a diverse range of illnesses, including sepsis, trauma, and regional ischaemia [9,14-21]. Recent studies suggest the prognostic value of hyperlactataemia is also aetiology dependent [22,23]. Hyperlactataemia is common in patients presenting with shock and is attributed to tissue hypoperfusion (type A lactic acidosis), with subsequent treatment focussing on increasing oxygen delivery with fluid, blood transfusion, and inopressor therapy [24,25]. There are many causes of hyperlactataemia that are not dependent upon anaerobic metabolism (type B lactic acidosis), including hepatic dysfunction, sympathetic stimulation, inadequate tissue oxygen extraction, thiamine deficiency, and medications [24-26]. In these cases, administering fluids and vasoactive medications may not benefit patients and risks harm from fluid overload [27-31].

An ED-based study identified different mortalities for type A and B hyperlactataemia, with type A associated with higher mortality than type B (45.8% vs. 12.5%) [32]. No analysis for the presence or absence of acidosis was performed. The authors were able to identify only one paper that explored the effects of lactate levels with or without acidosis on outcomes in an ED population [33]. This study suggested that the presence of acidosis as opposed to hyperlactataemia predicted mortality in sepsis.

The primary objective of this study was to investigate the effect of concurrent acidosis and hyperlactataemia on in-hospital mortality in undifferentiated adult patients presenting to the ED resuscitation area.

This article was previously posted to the Research Square preprint server on October 20, 2020.

## Materials and methods

Study design and setting

This was a single centre, retrospective observational cohort study carried out in a UK inner-city university teaching hospital. The authors analysed metabolic panel results from adult (≥17) patients who presented to the ED resuscitation area between February to May 2016 and September 2016 to March 2017. These patients received a venous blood gas (ABL 700 FLEX, Radiometer, Denmark) on arrival as a routine part of their initial assessment, along with recording basic physiology and a 12-lead electrocardiogram. In patients, intubated prehospital arterial blood gases were drawn initially. Blood gas data were downloaded directly and stored on a secure Microsoft Excel spreadsheet. In the case of multiple samples drawn during the same admission, only the first blood gas drawn was included in the analysis. Samples from patients with more than one presentation were included at each attendance. Unidentifiable data due to missing patient ID number, name and/or date of birth, and incomplete blood gas samples were excluded.

Variables

Lactate, pH, base excess (BE), and bicarbonate values were recorded. The medical record was inspected by the study team for in-hospital mortality and demographic data, including age, sex, and ethnicity. Data were anonymised in accordance with local data protection guidelines.

For this study, the authors selected a value of 2 mmol/L to define abnormality based on the Sepsis-3 consensus published in February 2016 [7]. Acidosis was defined as one or more of: pH < 7.35, base deficit < -3 mEq/L, or HCO_3_ < 20 mmol/L.

Groups

Results were stratified into six groups according to lactate value and the presence or absence of acidosis: normal lactate (0.0-2.0 mmol/L), intermediate lactate (2.1-4.0 mmol/L), and high lactate (>4.0 mmol/L), with and without acidosis. The primary outcome was in-hospital mortality. As a secondary outcome, the authors subdivided acidotic patients according to BE value to assess any correlation with in-hospital mortality. Additionally, the authors combined BE and lactate to assess their role in predicting mortality.

Statistical analysis

Continuous data are represented as means (standard deviation, SD) for normally distributed data or median (interquartile range) for non-normally distributed data. Categorical data are reported as number (percentage). Normality was checked using the Shapiro-Wilk test and by visually assessing the frequency distribution. Significance was set at p < 0.05. The difference in mortality between groups was calculated using odds ratio (OR) and are expressed with 95% confidence interval (CI) values.

Ethics

This study was deemed as research that did not require a formal REC review by the Trust Research and Clinical Governance Departments (in line with the UK Health Research Authority guidance) as there were no interventions, no deviations from usual care, and no identifiable data stored. The study was registered along local guidelines.

## Results

**Figure 1:**
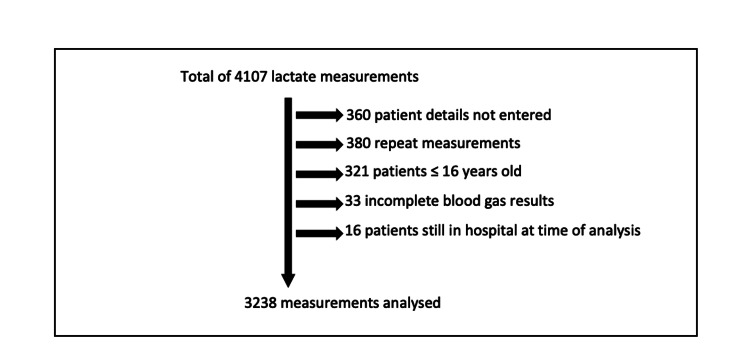
Participation flow diagram showing the number of samples excluded from the study.

Data were collected on 4,107 metabolic panel results and 3,238 were analysed (Figure 1). The mean age was 51 years and 59% were male. Of the 3,238 patients, 1,294 (40%) had a low lactate level (0.0-2.0 mmol/L), 1,429 (44.1%) had an intermediate lactate level (2.1-4.0 mmol/L), and 515 (15.9%) had a high lactate level (>4.0 mmol/L).

There was an overall in-hospital mortality of 5%. The intensive care unit (ICU) admission rate was 3.1% (data available on 1,882 patients for ward, ED, or ICU admission). Hyperlactataemia (and normal lactate ≤2 mmol/L) with acidosis was associated with increased mortality compared to hyperlactataemia (and normal lactate) without acidosis (Table 1). In patients with hyperlactataemia (>2.0 mmol/L), the presence of concurrent acidosis had a more significant effect on mortality in patients with high lactate (>4.0 mmol/L) than in patients with intermediate lactate (2.1-4.0 mmol/L) (Table 1). In the 2.1-4.0 mmol/L lactate group, mortality was 5.6% and 2.6% with and without acidosis, respectively (OR = 2.22; 95% CI = 1.28-3.85; p = 0.0045). In those with lactate >4.0 mmol/L, mortality was 19.3% and 3.9% in the groups with and without acidosis, respectively (OR = 5.85; 95% CI = 2.31-14.81; p = 0.0002) (Table 1).

**Table 1:** Groups stratified by lactate and acidosis showing mortality figures.

Lactate (mmol/L)	Mortality (%)	
	Acidosis	No acidosis
0.0-2.0	4.3	0.6
2.1-4.0	5.6	2.6
>4.0	19.3	3.9

Irrespective of lactate level, in-hospital mortality was 8.7% and 1.8% in the groups with and without acidosis, respectively (OR = 5.16; 95% CI = 3.48-7.65). Stratifying patients by lactate level, mortality was 6.9% and 2.1% in the patient groups with and without hyperlactataemia (>2.0 mmol/L), respectively (OR = 3.50; 95% CI = 2.32-5.33).

The authors combined BE and lactate to assess their role in predicting in-hospital mortality. Table 2 shows the results of these analyses. Combining lactate >4 mmol/L with BE < -6 gave a mortality rate of 31.9% in our cohort, 63 out of 197 patients died (OR = 14.0; 95% CI = 9.77-20.11). Using a combination of BE and lactate is the most specific indicator for in-hospital mortality. It conveys the highest positive predictive and negative predictive values of the four combinations analysed in this study.

**Table 2:** Statistical analyses of BE, lactate, acidosis, and systolic blood pressure as prognostic indicators for in-hospital mortality. Blood pressure data only available for 1,395 patients. Sens: sensitivity; Spec: specificity; PPV: positive predictive value; NPV: negative predictive value; BE: base excess; Lac: lactate; BP: blood pressure

	Sens (%)	Spec (%)	PPV	NPV
BE < -3	63	82	16	98
BE < -6	48	92	25	97
Lac > 4 BE < -6	39	96	32	98
Lac > 4 acidotic	46	90	19	97
Systolic BP ≤90 mmHg	18	96	19	95

## Discussion

This study suggests that in an undifferentiated cohort of adult ED patients treated in the resuscitation area, hyperlactataemia with co-existing acidosis conferred a higher risk of in-hospital death than elevated lactate levels alone (no acidosis). The margin was greater at higher lactate levels (>4 mmol/L compared to 2.1-4.0 mmol/L).

In keeping with previous studies, the authors found that the mortality rate of individuals with a lactate level of ≥2 mmol/L was higher than those with <2.0 mmol/L [4,21,23,34]. However, data also suggest the risk of in-hospital mortality to be higher in patients with acidosis, regardless of lactate level, than patients with no acidosis.

The authors report mortality of 2.1% for patients with a lactate level of ≤2.0 mmol/L, 3.8% with a lactate level of 2.0-3.9 mmol/L, and 15.5% with a lactate level of >4.0 mmol/L. The OR death for a lactate level of 2.03.9 mmol/L was 2.2 and for >4 mmol/L was 5.9. The blood gas samples were drawn during the initial assessment of adult patients treated in the resuscitation area prior to in-ED treatment within minutes of arrival. Previous ED-based studies have targeted populations selected by clinician concern or by diagnosis. Previous studies also involved blood samples drawn at variable times in the patients’ ED journey. In 2015, Datta et al. [21] reported a cohort of 747 undifferentiated (Scottish) ED patients (15% admitted to critical care, median age 67, 27,500 patients attended during the study period, 2.7% had blood gas sampled, samples drawn less than four hours of ED arrival) with arterial lactate values of <2, 2.0-3.9, and ≥4.0 mmol/L associated with 30-day mortalities of 10.1%, 19%, and 50%, respectively. Blood gas analysis was performed at the discretion of the treating clinician, suggesting that sicker patients may have been targeted. Contenti et al. [35] reported (94% venous) lactate measurement in 11% (13,089/118,737 adults, median age 52, time of lactate sample not reported) of attendees at a French ED (increasing to around half if the diagnosis was an infection). The authors did not report mortality stratified by lactate level. van den Nouland et al. [32] reported lactate-related mortality in 5.8% (1,144/19,822) of the patients admitted to (Dutch) medical wards (median age 67, time of blood sampling not stated). The authors found that lactate levels of <4 mmol/L and >4 mmol/L were associated with mortalities of 18.5% and 40.6%, respectively. Patients who did not have lactate measured had a 28-day mortality of 9.5%. In this study, patients had a median age of 51 years and samples were drawn prior to treatment, which may explain why the reported mortality rates were lower than those in these clinician-selected groups.

van den Nouland et al. [32] and Datta et al. [21] reported arterial lactate levels whereas this study and Contenti et al. [35] predominantly included venous samples. This may alter the reported risk associated with lactaemia as venous samples overestimate lactate levels compared to arterial samples in the ED setting [36], with the discrepancy more marked at higher levels. High levels of correlation between venous and arterial lactate levels [4] have been reported, but in these studies, the majority of samples were in the normal range. Bloom et al. [36] investigated the agreement between venous and arterial samples at pathological lactate levels and noted increasing disagreement with increasingly elevated lactate levels. This work suggests around 17% of the patients with a lactate level of >4.0 mmol/L by venous sampling would have arterial levels below this, and 36% of patients with a venous lactate level of >2.0 mmol/L would have arterial lactate of <2.0 mmol/L. This would see a higher proportion of low-risk patients identified by venous as opposed to arterial samples. However, three large international sepsis trials used venous or arterial lactate as their inclusion criteria to define shock.

Pedersen et al. [23] studied a cohort of 5,360 adult undifferentiated ED patients who received an arterial or venous blood gas (proportion not reported) within four hours of admission (exact times not reported). In this study, 77.2% patients had a lactate level of <2 mmol/L, 16.2% 2.0-3.9 mmol/L, and 6.6% >4 mmol/L. In this study, the authors identified 40.0% of the patients to have a lactate level of ≤2.0 mmol/L, 44.1% 2.1-4.0 mmol/L, and 15.9% >4.0 mmol/L. Samples in this study were drawn on arrival prior to any treatment being delivered, which may account for the increased proportion of elevated lactate levels compared to Pedersen et al. Pedersen et al. [23] reported (seven-day) mortality rates of 2.9%, 7.8% (OR death 3.0), and 23.9% (OR death 11.5) for patients with low (0-1.9 mmol/L), intermediate (2-3.9 mmol/L), and high lactate (≥4 mmol/L), respectively, which are slightly higher than those reported in this study. The authors investigated various diagnostic subgroups and reported lactate to be a useful prognostic biomarker for patients with a diagnosis of infection, trauma, cardiac, and gastrointestinal disease, but not (or of uncertain value) for patients with neurological, non-infective respiratory, endocrine diseases, alcohol intoxication, or malignancy. The study reported here did not set out to look at subgroups that would be too small for meaningful comparison and the authors had no means to assess for the diagnostic accuracy reported in the medical records.

The authors are aware of only one small study that previously described the effect of acidosis on lactataemia and mortality. Lee et al. [33] investigated 126 patients with severe sepsis or septic shock with similar aims as this study and similar findings that acidosis was associated with a higher risk of death than lactataemia in isolation. Patients with hyperlactataemia alone (lactate ≥2 mmol/L, no acidosis) had similar mortality rates compared to patients with normal pH and lactate levels. However, in-hospital mortality was significantly higher in patients with lactic acidosis compared to those with normal pH and lactate. The authors concluded that the acid-base status of patients should be considered when using tests such as lactate to predict outcomes in patients with sepsis.

In this study, the combination of a lactate >4 mmol/L and acidosis had a sensitivity of 46% and specificity of 90% for in-patient mortality, while lactate >4 mmol/L combined with BE <-6 was associated with a sensitivity of 39% and specificity of 96%. Figures for BE <-6 alone were 48% and 92%, respectively. In an ICU-based study, Smith et al. [34] reported that the combination of BE <-4 mmol/L and lactate >1.5 mmol/L was more sensitive and specific for mortality than either alone, with a sensitivity of 80.3% and specificity of 58.7% for mortality. Husain et al. [37] retrospectively investigated the prognostic individual value of lactate and base deficit in a surgical ICU setting. The authors reported initial and 24-hour lactate (≥2 mmol/L) correlated well with mortality. Base deficit (<-2) only correlated with mortality in trauma patients at 24 hours and not on admission. These data suggest each of these is useful in identifying patients with high mortality. No acid-base/lactate combination was found to be sensitive enough to use as a screen for critical illness.

Limitations

As with all retrospective studies the authors were dependent on data entered into the patients’ clinical record by clinicians prior to the study being performed, over which no quality assurance was possible. A total of 360 (9%) samples were excluded from analysis as they did not have correct patient identifiable data (name, hospital number, date of birth). These blood gases were commonly very abnormal, presumably as these samples were taken from critically ill patients on their arrival in the ED and prior to booking them onto the computer system. Missing data is a common problem in retrospective studies on this topic, for example, Contenti et al. [35] reported 10% missing data.

The authors had originally planned to obtain blood gases for a consecutive 12-month period but were unable to download blood gas data for a three-month block. However, there is no data to suggest blood gas values vary with time of year so this is not likely to impact our analysis.

The primary outcome was in-hospital mortality, defined as whether the patient survived to discharge. This could be considered a less clinically valuable endpoint than seven-day or 30-day mortality as it did not take into account patients transferred from our institution to another, in which they could have died. The study presented in this journal was performed on a single site limiting external validity, in particular, the mean patient age was younger than similar studies. The authors did not report findings for different diagnostic groups and work published since this study was designed to have identified that lactate levels offer different prognostic information for different patient groups [23]. Future work should include a large enough sample to assess different patient groups by diagnosis, treatment, and disposition.

The authors did not explore the effect of confounders on lactate levels, such as alcohol use, liver disease, and chronic illness. These may impact lactate levels during the acute phase of the illness. However, these factors may not be available or are unreliably available to clinicians during the early phase of resuscitation. The authors also did not detail prehospital care delivered. However, the hospital serves a central inner-city population with short ambulance transport times. Previous local work (unpublished) has shown almost all patients receive under 500 mL intravenous fluid prior to arrival at the ED. Finally, the authors did not adjust findings for confounding variables such as blood pressure or age or the presence of chronic illnesses associated with increased mortality.

## Conclusions

The results of this study suggest blood gas results obtained on presentation to the ED are a useful prognostic marker. Lactataemia associated with acidosis is a more accurate predictor of in-hospital mortality than elevated lactate alone. The effect of coexisting acidaemia varies with the severity of hyperlactataemia. The marked differences in mortality associated with hyperlactataemia with and without acidosis have significant implications for the role of lactataemia in identifying critical illness and as a resuscitation endpoint. A combination of BE <-6 and lactate >4 mmol/L was associated with the highest specificity for mortality.

The authors believe this to be the first study to publish data on acidosis and lactaemia in undifferentiated patients presenting to the ED. Further studies examining the effect of lactate and various measures of acid-base disturbance in different subgroups of ED patients are required.
